# Research progress of LINE-1 in the diagnosis, prognosis, and treatment of gynecologic tumors

**DOI:** 10.3389/fonc.2023.1201568

**Published:** 2023-07-20

**Authors:** Jiaojiao Fu, Tiansheng Qin, Chaoming Li, Jiaojiao Zhu, Yaoyao Ding, Meiying Zhou, Qing Yang, Xiaofeng Liu, Juanhong Zhou, Fan Chen

**Affiliations:** ^1^ The First Clinical Medical College of Gansu University of Chinese Medicine, Gansu Provincial Hospital, Lanzhou, China; ^2^ Department of Obstetrics and Gynecology, Gansu Provincial Hospital, Lanzhou, Gansu, China; ^3^ The First Clinical Medical School, Lanzhou University, Lanzhou, Gansu, China; ^4^ National Health Commission (NHC) Key Laboratory of Diagnosis and Therapy of Gastrointestinal Tumor, Gansu Provincial Hospital, Lanzhou, Gansu, China; ^5^ The First People’s Hospital of Longnan, Longnan City Hospital, Longnan, Gansu, China

**Keywords:** gynecologic cancers, retrotransposons, diagnostic, therapy, prognosis

## Abstract

The retrotransposon known as long interspersed nuclear element-1 (LINE-1), which is currently the sole autonomously mobile transposon in the human genome, can result in insertional mutations, chromosomal rearrangements, and genomic instability. In recent years, numerous studies have shown that LINE-1 is involved in the development of various diseases and also plays an important role in the immune regulation of the organism. The expression of LINE-1 in gynecologic tumors suggests that it is expected to be an independent indicator for early diagnosis and prognosis, and also, as a therapeutic target, LINE-1 is closely associated with gynecologic tumor prognosis. This article discusses the function of LINE-1 in the diagnosis, treatment, and prognosis of ovarian, cervical, and endometrial malignancies, as well as other gynecologic malignancies. It offers fresh perspectives on the early detection of tumors and the creation of novel anti-tumor medications.

## 1 Introduction

Gynecological tumors represent a major threat to women’s health, including ovarian, cervical, and endometrial cancers. With a five-year survival rate of 45 percent, ovarian cancer has the highest mortality rate among gynecological cancers, trailing only cervical and endometrial cancer in its incidence (1). Cervical cancer is the most commonly diagnosed cancer and the fourth leading cause of death in women, according to statistics, with an estimated 604,000 new cases and 342,000 fatalities worldwide in 2020 (2). Additionally, endometrial cancer, the sixth most common cancer in women, has seen a 132% increase in the overall incidence over the past 30 years, and the associated mortality rate is increasing rapidly (3). As a result, there is a critical need for efficient early diagnostic methods to find gynecological malignancies.

Transposons, also known as “jumping genes”, are mobile (Deoxyribonucleic acid) DNA sequences in the genome that compose approximately 45% of the human genome (4). According to their transcribing methods, there are two primary categories: The first category is DNA transposons, which use a “cut-and-paste” method to transport DNA sequences from one location to another after specifically recognizing them by their transposases. The second type is RNA transposons, sometimes referred to as retrotransposons (RT), which take DNA sequences as templates and first produce intermediate forms of mRNA before producing cDNA through the activity of reverse transcriptase and then “paste” the DNA sequence to a new spot (5). Retrotransposons are divided into long terminal repeat (LTR) and non-long terminal repeat (Non-LTR) elements. Non-LTR mainly includes long scattered repeat element LINE-1, short scattered repeat element SINE and Alu (6). Among them, LINE-1, the most active type of Non-LTR transposon, is the only class of human reverse transcription transposon with autonomous activity, which can cause genomic instability and is considered a harmful endogenous substance with potential carcinogenic activity (7).

Studies have shown that LINE-1 plays a role in gynecologic malignancies, such as ovarian, cervical, and endometrial cancers, so it is important to investigate the role of LINE-1 in the early diagnosis and prognosis of gynecologic tumors. In this paper, we review the composition and regulatory mechanism of LINE-1 and its role in the early diagnosis, prognosis, and treatment of ovarian, cervical, and endometrial cancers, which will provide a theoretical basis for the development of novel tumor molecular markers or targeted medications.

## 2 Introduction of LINE-1

### 2.1 Composition of LINE-1

**Figure 1:**
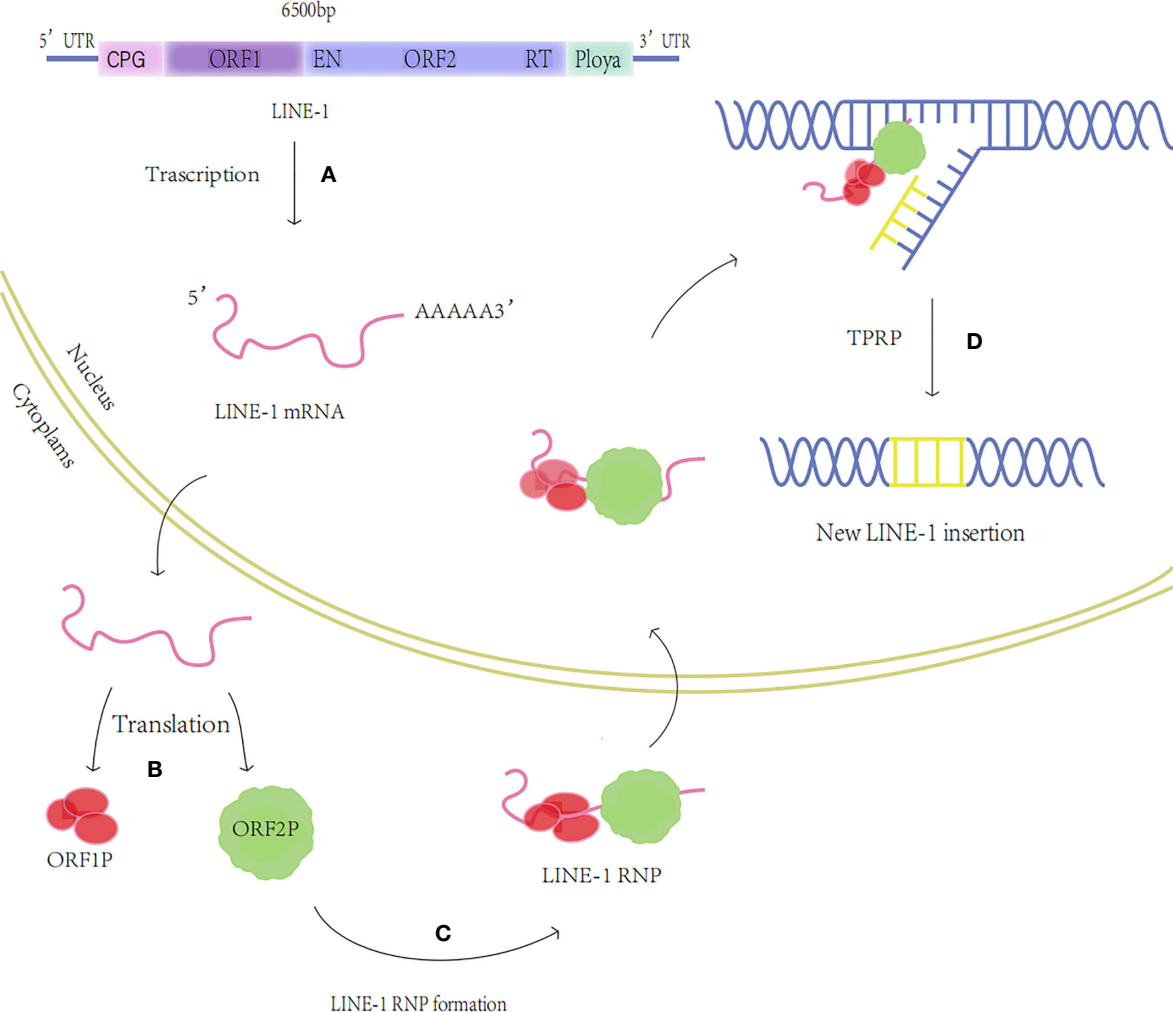
The transposition process of LINE-1. **(A)** Transcription: The LINE-1 mRNA is produced. **(B)** Translation: Proteins ORF1p and ORF2p are produced. **(C)** LINE-1 RNP formation: ORF1p and ORF2p bind to LINE-1 RNA to generate LINE-1 RNP. **(D)** TPRT: The nucleic acid endonuclease activity and reverse transcriptase activity of ORF2p insert LINE-1 into the new locus through the TPRT process.

The LINES family includes LINE-1, LINE-2, and LINE-3, of which the most abundant and functionally rich is LINE-1 with approximately 500,000 copies, accounting for 17% of the human genome (8). About 100 of these are still complete and functioning, while the majority have lost retrotransposition owing to deletion, mutation, or truncation (4, 9). The full-length LINE-1 sequence is 6000-7000 base pairs (bp) and contains a 5’ untranslated region (UTR), two open reading frames (ORF) ORF1 and ORF2, and a 3’ UTR containing a polyadenylation signal (polyA) (10). LINE-1 activity is driven by RNA polymerase II binding to the 5’UTR internal promoter, and ORF1 and ORF2 encode two proteins required for reverse transcription, ORF1p, and ORF2p, respectively, ORF1p is an RNA-binding protein with nucleic acid chaperone activity (11); ORF2p contains nucleic acid endonuclease (EN) and reverse transcriptase (RT) structural domains (12). Among them, the endonuclease cleaves genomic DNA by specifically recognizing the 5’-TTTT/AA-3’ sequence, and its induced single-stranded DNA break triggers the recruitment of poly(ADP-ribose) polymerase 2 (PARP2) at the LINE-1 integration site to promote reverse transcriptional translocation (13). Reverse transcriptase activity can use LINE-1mRNA as a template to produce cDNA (14). ORF1p and ORF2p are translated in the cytoplasm and first bind to LINE-1 mRNA to form the LINE-1RNA/ORF1p/ORF2p ribonucleoprotein (RNP) complex particle (15), RNP is then transported into the nucleus using the membrane-associated nuclear endosome sorting complex (ESCRT) required for transport (16), Generation of a new copy of LINE-1 and its insertion into host DNA through a unique mechanism of target-primed reverse transcription (TPRT) (17) (**Figure 1**). Additionally, LINE-1 can assist in transposing components without an independent reverse transcription function, such as SINE and ALU (18).

### 2.2 Regulation of LINE-1

LINE-1 played an essential role in the evolution of the species, however, in normal somatic cells, the host cell has tight control over LINE-1 translocation to maintain genomic stability (**Table 1**). The 5’UTR promoter region of LINE-1 is rich in guanine and cytosine enriched regions (cytosine phosphate-guanosine, CpG) (28), which are often repressed by the organism in normal tissues, while the absence or deficiency of methylation, i.e. CPG hypomethylation, enhances its reverse transcription activity and promotes retrotranscription transposition (29). Sanchez-Luque et al. reported a conserved Yin-Yang 1 (YY1) transcription factor binding site that mediates LINE-1 promoter methylation in pluripotent and differentiated cells, thereby inhibiting LINE-1 reverse transcriptional translocation (19). N6-methyladenosine (m6A), the most prevalent methylation modification in eukaryotic mRNA, promotes LINE-1 RNA expression and reverse transcriptional translocation, enhances translation efficiency, and promotes RNP formation (20, 21). In addition, oncogenes and tumor suppressor genes are also involved in the regulation of LINE-1. Myc oncoprotein may be a major regulator of LINE-1 transcription during cancer development, Sun et al. found that Myc expression levels significantly and inversely correlated with LINE-1 expression in breast and ovarian tumors (22). Tumor suppressor P53, which inhibits LINE-1 in cancer tissues derived from human cell cultures by acting on the 5’UTR promoter of LINE-1 and stimulating local deposition of inhibitory histone marks, limits autonomous replication of mobile elements in cells (23). Mita et al. revealed that the tumor suppressor BRCA1, which is involved in DNA homologous recombination repair (HR), has a strong inhibitory effect on LINE-1 retrotransposition, and in the cytoplasm, BRCA1 inhibits ORF2p translation by binding to its mRNA (24). Therefore, LINE-1 expression and its activity in an environment of HR dysregulation may increase genomic instability and accelerate tumor development or the formation of drug-resistant clones after treatment. In addition, endogenous proteins also regulate the expression of LINE-1. Liang et al. showed that The apolipoprotein B mRNA-editing catalytic polypeptide 3 APOBEC3 (A3) family, APOBEC3 (A3DE) inhibited LINE-1 reverse transcription translocation by interacting with ORF1p and affecting LINE-1 reverse transcriptase activity (25). Dual specificity protein phosphatase 1 (DUSP1), is involved in the negative regulation of cell proliferation and suppression of inflammatory responses, and its activity downregulates LINE-1 in cancer cells (26). The human silencing center (Hush) complex, a newly discovered epigenetic complex, represses LINE-1 expression in the organism, selectively binds evolutionarily young full-length LINE-1 and promotes the deposition of histone H3Lys9 trimethylation (H3K9me3) and the formation of heterochromatin for transcriptional silencing (27, 30–32).

**Table 1:** Regulation of LINE-1.

Form of regulation	Participating substances	Mechanism	Effect on LINE-1	References
Methylation Modification	Transcription factor binding site(YY1)	Hypomethylation	Promoting LINE-1 expression	(19)
	N6-methyladenosine (m6A)	Methylation	Inhibition of LINE-1 reverse transcription	(20, 21)
Oncogenes	Myc oncoprotein		Inhibition of LINE-1	(22)
Tumor suppressor	TP53	Inhibitory histone deposition	Inhibition of LINE-1 expression	(23)
	BRCA1	Binding LINE-1 mRNA	Inhibition of ORF2p translation	(24)
Endogenous proteins	APOBEC3(DE)	Combined with ORF1p	Inhibition of LINE-1 reverse transcriptase activity	(25)
	DUSP1		Down-regulation of LINE-1 expression	(26)
Epigenetic complexes	Hush	Promotion of H3K9me3 deposition and heterochromatin formation	Repression of LINE-1 transcription	(27)

## 3 LINE-1 and cancer

### 3.1 Impact of LINE-1 hypomethylation on cancer

A frequent epigenetic hallmark of cancer is genome-wide hypomethylation, and LINE-1 hypomethylation can be utilized as a marker of genome-wide hypomethylation (33, 34). LINE-1 hypomethylation increases LINE-1 mRNA expression, activates reverse transcription activity, and causes insertional mutations, rearrangements, and chromatin mutations in genomic DNA (7), which ultimately lead to tumorigenesis (35). Studies have shown that LINE-1 hypomethylation is involved in the early development of various cancers and plays a role in cancer proliferation and invasion. Hoshimoto et al. found that overall DNA methylation levels were remarkably lower in esophageal squamous cell carcinoma (ESCC) than in normal mucosa, suggesting that LINE-1 hypomethylation may be an early event in ESCC and significantly correlated with the depth of tumor invasion in primary ESCC (36). Another study also confirmed that LINE-1 hypomethylation promotes aggressive tumor behavior by causing genomic gain of oncogenes such as cell cycle protein-dependent kinase 6 (CDK6) in esophageal squamous carcinoma (37). In addition, LINE-1 hypomethylation is closely associated with cancer prognosis, and studies have shown that LINE-1 hypomethylation is related to poor prognosis in lung adenocarcinoma (38), gastric cancer (GC) (39), and colorectal cancer (40), and also can be used as a diagnostic marker for colorectal cancer (40). In studies of lung adenocarcinoma, LINE-1 methylation levels were shown to be negatively correlated with Ki-67 expression, which reflects the aggressive, proliferative nature of cancer, and therefore, lower overall methylation levels in cancer may induce an increased proliferative capacity of the tumor (38). Interestingly, hepatocellular carcinoma (HCC) also exhibits global DNA hypomethylation which increases genomic instability and rearrangements in HCC, allowing LINE-1 to insert into the c-MET gene and drive its transcription through a “copy-and-paste” mechanism, known as L1-MET, ultimately promoting the oncogenic pathway in HCC and leading to poor prognosis (41, 42). Recently, by applying new computational tools and long-read nanopore sequencing to directly infer the CpG methylation of new and existing transposable element insertions in hippocampal heart and liver and paired tumor and non-tumor livers, Ewing et al. found significant demethylation of young LINE-1 retrotransposons in cancer (43). In addition, Park et al. showed that LINE-1 methylation levels could also be used as a good early screening indicator to distinguish healthy individuals from those with lung and breast cancer (44). In summary, LINE-1 hypomethylation is closely associated with the development of various malignancies and is expected to be an independent biomarker of early diagnosis and poor prognosis of tumors.

### 3.2 ORF1p and ORF2p expression as diagnostic markers for cancer

LINE-1 mRNA translates two proteins required for reverse transcription, ORF1p, and ORF2p, and their overexpression is a biomarker and prognostic correlate for a variety of tumors. Early studies have shown that LINE-1 ORF1p is barely detectable in normal human cells (45), but it is expressed in cancers such as ovarian (46), prostate (47), bladder (48), lung (49), and esophageal cancers (50), and can be used as a diagnostic marker for cancer. Later, Ardeljan et al. examined ORF1p expression in 22 colorectal cancer tissues by immunohistochemistry and showed that all tumors were positive (51). In another study, LINE-1 ORF1p was highly expressed in p53 mutated cancers and was associated with the tissue type of advanced gynecologic tumors (52). Furthermore, its expression suggests a poor cancer prognosis. Studies in breast cancer have shown that nuclear localization of ORF1p protein is significantly associated with poorer survival (53). However, ORF2p is difficult to detect in tumors (54). The RT encoded by LINE-1-ORF2 has the potential to exert tumor-promoting effects at the epigenetic level. Sciamanna et al. proposed a model in which LINE-1-RT drives a previously unrecognized global regulatory process that exerts global epigenetic regulation of the cellular transcriptome by intercepting RNA and reverse transcribing it into RNA: DNA hybrids. LINE-1-RT dysregulation drives cellular transformation and tumorigenesis, suggesting that ORF2p may have an impact on cancer cell heterogeneity (55). De Luca et al. investigated the expression and localization of ORF2p in human cancer cells and tissues by a highly specific monoclonal antibody (mAb chA1-L1) and found that LINE-1 ORF2p was highly expressed in transformed cell lines and staged epithelial cancer tissues (colon, prostate, lung, and breast), but not detected in untransformed cells, and they also found that ORF2p was overexpressed in precancerous tissues of colon and prostate, thus ORF2p can be considered as a potential early diagnostic biomarker (56).

### 3.3 LINE-1 regulates oncogene expression in cancer

Numerous studies have shown that LINE-1 insertion in somatic cells can induce cancer development through the activation of proto-oncogenes. In a study on breast cancer, LINE-1 insertion was found to cause rearrangement and amplification of the MYC oncogene, leading to the development of ductal adenocarcinoma of the breast (57). Later, Wolff et al. found that LINE-1 hypomethylation activates the MET oncogene in bladder cancer, leading to the activation of surrounding genes that can promote tumorigenesis through synergistic effects (58). Similarly, LINE-1 can ultimately induce cancer by suppressing the expression of tumor suppressor genes. LINE-1 retrotransposition can affect local immune homeostasis by interfering with the expression of the tumor suppressor gene FGGY in squamous lung cancer and disrupting cellular energy metabolism, leading to tumor progression and poor prognosis (59). In addition, earlier studies characterizing reverse transcription transposon insertions in the whole genome of colorectal cancer in 202 cases identified highly variable reverse transcription transposon activity, and insertion of LINE-1 in the exon of the tumor suppressor gene APC was detected in approximately 1% of cases, which may be a tumor-initiating event and was associated with low survival (60, 61). Finally, Rodriguez-Martin et al. through a pan-cancer whole genome analysis (PCAWG), found that aberrant integration of LINE-1 retrotransposons plays an important role in reshaping the structure of the cancer genome, mainly through deletion of base regions on chromosomes, which leads to massive deletion of tumor suppressor genes or triggers amplification of oncogenes, and complex ectopics to promote cancer progression (62).

## 4 LINE-1 with a role in gynecologic malignancies

**Figure 2:**
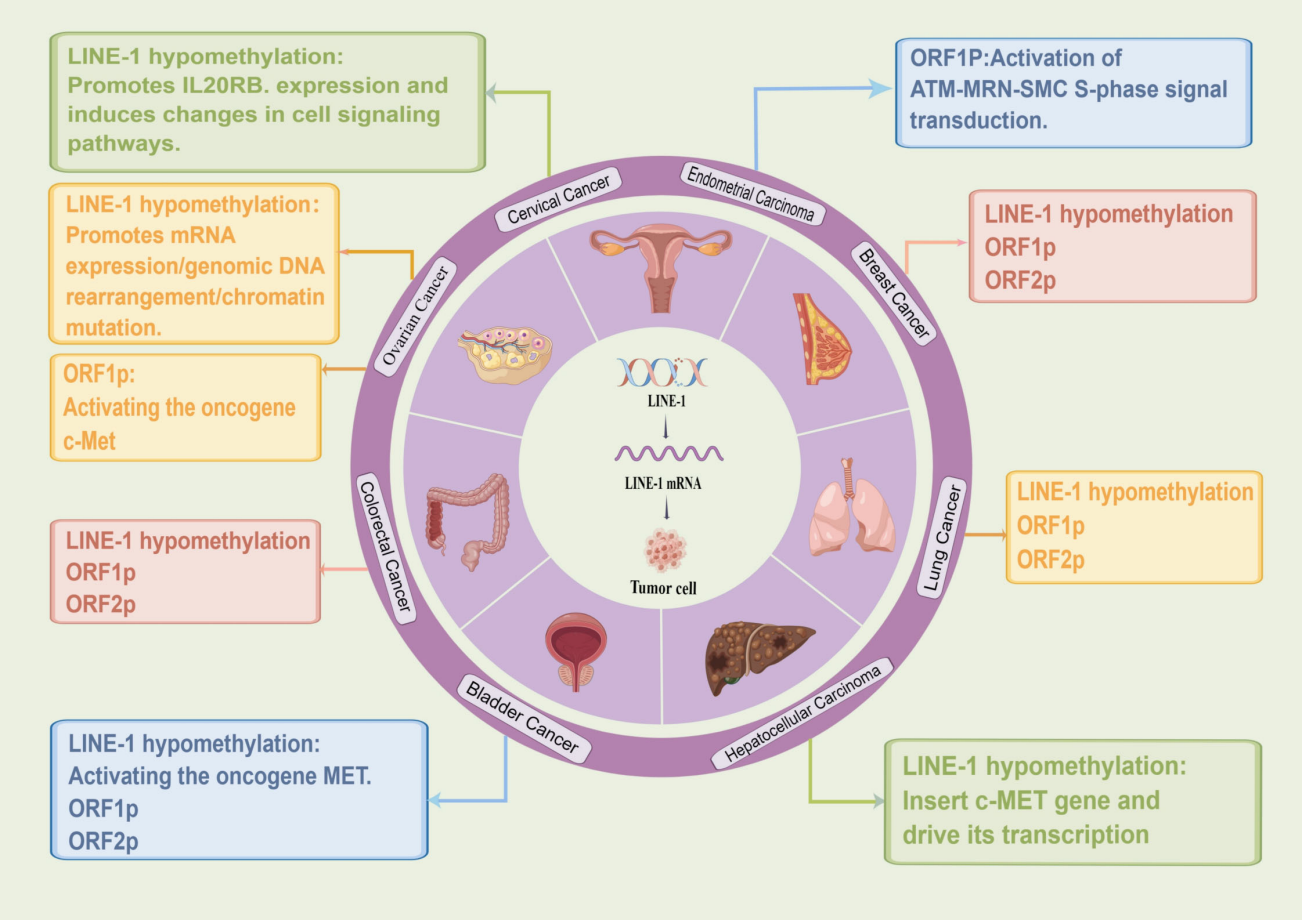
Role of LINE-1 in different tumors. (By Figdraw.).

In a variety of tumors, including gynecologic tumors, LINE-1 is involved in tumor development through the expression of its mRNA, promoter hypomethylation, and its reverse transcriptional production of OPRF1p and OPRF2p (**Table 2**) (**Figure 2**).

**Table 2:** LINE-1 translocation and related gynecologic tumors.

Tumor prognosis, diagnostic markers	Mechanism	Related cancer	References
LINE-1 mRNA	Acquired insertion of LINE-1 enhances STC1 oncogene expression	Ovarian cancer (HGOSC)	(63)
LINE-1 hypomethylation	Promotes mRNA expression/genomic DNA rearrangement/chromatin mutation	Ovarian cancer (OEA, COCC)	(64)
	Promotes IB20 expression and induces altered signaling pathways and increased cell invasiveness	Cervical Cancer	(65)
	Mutation, amplification, and deletion of genomic DNA	Gestational Trophoblastic Neoplasms (GTN)	(66)
ORF1p	Activates c-Met proto-oncogene expression and promotes tumor progression	Ovarian cancer (STIC)	(52)
	Activates ATM-MRN-SMC S-phase signaling and causes DNA damage	Endometrial cancer	(67)
	Insertion mutations, modification of regulatory sequences, causing genomic instability	differentiated Vulvar Intraepithelial Neoplasia (dVIN)	(68)

### 4.1 LINE-1 with a role in ovarian cancer

Ovarian cancer is one of the three major malignant tumors of the female reproductive system and has the highest mortality rate among gynecologic tumors because it is asymptomatic and resistant to chemotherapy in its early stages. According to its histopathological characteristics, ovarian cancer is mainly divided into three categories: epithelial ovarian cancer(EOC), germ cell tumors, and gonadal-mesenchymal tumors. Among them, EOC is the most common cause of death (69), and its pathological type is most frequently High-Grade Serous Ovarian Cancer(HGOSC). Numerous studies have shown that LINE-1 can be used as a molecular marker for early diagnosis and prognosis of ovarian cancer. Pattamadilok et al. assessed the genome-wide LINE-1 methylation status in isolated cell populations by an improved quantitative combined bisulfite restriction analysis(COBRALINE-1) PCR technique and found that LINE-1 hypomethylation was an early event in the development of EOC. The Cox regression model showed that excessive LINE-1 hypomethylation shortened overall survival (70). Similarly, another study identified LINE-1 expression as an early event in HGOSC carcinogenesis that may precede the development of ovarian cancer itself (71). In addition, Tang et al. detected LINE-1 acquired insertions in HGOSC with BRCA1 genetic mutations by two novel techniques, next-generation sequencing (TIPseq) and machine learning-based computational pipeline (TIPseqHunter), suggesting that LINE-1 is a major source of heritable structural variants in the human genome (72). Acquired chemoresistance is a major driver of mortality in HGOSC patients, and Nguyen et al. analyzed the presence of LINE-1 hypomethylation and full-length transcripts in HGOSC. Meanwhile, they also identified a tumor-specific LINE-1 insertion that enhanced the expression of the STC1 oncogene, thereby increasing *in vitro* chemoresistance, suggesting that the LINE-1 insertion was associated with prognosis (63). In addition, Senthong et al. found that LINE-1 hypomethylation is an early molecular event involved in the malignant transformation of ovarian endometrioid adenocarcinoma (OEA) and ovarian clear cell carcinoma (COCC) and can be used as a basis for diagnostic biomarkers (64). The same conclusion was later reached by Xia et al. who found that TTC28-L1-mediated transduction occurs early in the development of CCOC and endometriosis-associated ovarian cancers (EAOCs) (73). Besides, investigators found that LINE-1 ORF1p was observed in a wide range of ovarian cancer specimens and was present independently of human epididymal protein 4(HE4) and cancer antigen 125(CA-125), and they confidently detected ORF1p expression in biological fluids including ascites and plasma from ovarian cancer patients using Immuno multiple reaction monitoring-mass spectrometry (iMRM-MS) (74). LINE-1 ORF1p significantly activates the expression of c-Met proto-oncogene in ovarian cancer, thus participating in tumor progression (46). Xia et al. by tissue microarray (TMA’s) technique, found high expression of ORF1p in P53-mutated plasmacytoid intraepithelial carcinoma (STIC), suggesting that ORF1p may be clinically useful as a diagnostic immunohistochemical marker for P53-deficient STIC lesions (52). Furthermore, Yun et al. examined the expression of LINE-1ORF1p in 100 ovarian cancer tissues and found that ORF1p expression in ovarian cancer was higher in metastatic disease and elderly patients (75). It is suggested that LINE-1-ORF1p has the potential to be used as a diagnostic marker for ovarian cancer and its metastatic potential. Thus, evidence suggests that LINE-1 hypomethylation and ORF1p can be used as biomarkers for early diagnosis of ovarian cancer and are associated with prognosis.

### 4.2 LINE-1 with a role in cervical cancer

Cervical cancer is mainly caused by human papillomavirus HPV16 and HPV18 infection, and LINE-1 hypomethylation plays an important role in the progression of cervical cancer. Smith et al. used combined bisulfite restriction analysis and PCR techniques to measure LINE-1 hypomethylation levels in squamous cell carcinoma (SCC) and carcinoma *in situ* (CIS) of the uterine cervix and found a significant correlation between the degree of LINE-1 hypomethylation and progression of cervical mucosa from normal to elevated CIS to invasive cancer (76). In addition, it has also been found that LINE-1 methylation status in white blood cell DNA may be a cost-effective biomarker for high-grade cervical intraepithelial neoplasia (CIN2+) in high-risk human papillomavirus (hrHPV)-positive women (77), suggesting that LINE-1 methylation status may be used as a non-invasive early diagnosis in women at risk of cervical cancer. In further, Flatley and his colleagues found that most cervical cancer samples had global DNA hypomethylation compared to precancerous lesions (78). Later, Curty et al. also reported LINE-1 expression in cervical cancer and found that a comparison between HPV co-infection and HPV single infection showed a higher percentage of differential LINE-1 expression than the rest (65). In addition, LINE-1 hypomethylation also promotes IL20RB expression to induce alterations in cellular signaling pathways, leading to abnormal cell proliferation and increased tumor cell invasiveness. They also found that DNA methyltransferase 1 (DNMT1) in cervical cancer was significantly correlated with up- and downregulation of LINE-1 expression, and DNMT1 overexpression led to transcriptional repression of genes through hypermethylation, suggesting that LINE-1 correlates with endogenous factors (such as IL20 family genes and DNMT1) and exogenous factors (HPV) in cervical cancer. Recently, Sun et al. (79) used bisulfite LINE-1 pyrophosphate sequencing and ELISA-based methods to analyze global DNA methylation and showed a progressive global DNA methylation reduction from normal cervical cells to cervical cancer samples. In conclusion, LINE-1 is closely associated with the development of cervical cancer and can be used as an early screening indicator for cervical cancer.

### 4.3 LINE-1 with a role in endometrial cancer

Endometrial cancer is an epithelial malignancy originating from the endometrium. Compared to other gynecologic tumors, LINE-1 has been less studied in endometrial cancer, but some studies have indicated that LINE-1-ORF1p is associated with endometrial cancer. McKerrow et al. found that in endometrial cancer, upregulation of LINE-1-ORF1p expression led to increased RAD50-S635 phosphorylation and activation of ATM-MRN-SMC S-phase signaling, resulting in a DNA damage response as well as replication stress not directly attributable to LINE-1 insertion (67). In addition, LINE-1-ORF1p expression positively correlated with P53 mutation, copy number Alteration (CNA), and DNA replication initiation protein complex, and the expression of LINE-1-ORF1p was on average approximately two-fold higher in p53 mutant endometrial cancers. This indicates that LINE-1-ORF1p expression is positively correlated with structural genomic alterations in tumor tissue (67).

### 4.4 LINE-1 with a role in gestational trophoblastic neoplasms and differentiated vulvar intraepithelial neoplasia

GTN includes invasive staphyloma, choriocarcinoma, placental trophoblastic tumors, and epithelioid trophoblastic tumors. Although staphyloma can now be diagnosed earlier than before, the incidence of GTN after staphyloma remains unchanged. However, the correlation between malignant staphyloma and LINE-1 has rarely been studied. Lertkhachonsuk et al. (66) established a ROC curve for LINE-1 partial hypomethylation to predict whether postmenopausal GTN would occur, and they concluded that the decrease in partial methylation of LINE-1 occurs early before the clinical manifestation of malignant transformation and that choriocarcinoma and invasive staphyloma have higher levels of LINE-1 hypomethylation sites; therefore, partial methylation levels of LINE-1 may be a promising marker for monitoring staphyloma before progression to GTN.

Vulvar cancer is a relatively rare gynecologic cancer, and Hofstetter et al. (68) used immunohistochemistry to determine LINE-1-ORF1p and p53 expression in dVIN, suggesting that ORF1p may be a useful diagnostic marker for dVIN, especially if wild-type p53 is preserved.

## 5 LINE-1 as a therapeutic target for gynecologic tumors

### 5.1 Targeted epigenetic therapy

#### 5.1.1 Targeting LINE-1 hypomethylation

DNA methylation and histone modifications are the main forms of epigenetic modifications, and studies have shown that genome-wide LINE-1 hypomethylation is associated with immune escape features in aneuploid tumors, promotes high mutations and high chromosomal copy number changes, and is an important manifestation of epigenetic abnormalities in a variety of tumors (80). Studies related to ovarian cancer have shown that LINE-1 hypomethylation directly activates the ATM-MRN-SMC S-phase checkpoint pathway by promoting mRNA expression, genomic DNA rearrangements, and chromatin mutations, leading to double-strand breaks (DSBs) and replication stress, which ultimately cause cancer development (67). In addition, LINE-1 hypomethylation promotes IL20 expression in cervical cancer, induces altered signaling pathways, and increases cell invasiveness, which is an important epigenetic alteration in several cancers, including gynecologic tumors (65). Therefore, inhibition of LINE-1 hypomethylation is an effective therapeutic strategy. In a clinical trial, LINE-1 hypomethylation was found to be associated with the progression of oral precancerous lesions to head and neck squamous cell carcinoma (HNSCC), and short-term soy isoflavone intake resulted in a significant increase in tissue-specific overall methylation in patients, suggesting that patients’ LINE-1 hypomethylation levels could be modulated by soy isoflavone supplementation. Also, the association of LINE-1 hypomethylation with genetic instability, tumorigenesis, and prognosis suggests that soy isoflavones may be used as potential cancer-preventive agents (81). Szabo et al. also found that olive oil (EVOO) improved the hypomethylation of LINE-1 reverse transcription transposon DNA in an environmental carcinogen model compared to controls, resulting in reduced tumor incidence (82). Therefore, it can be considered that the treatment of gynecological tumors by targeting and regulating LINE-1 hypomethylation. In addition, DNA methyltransferase (DNMT), an important epigenetic molecule that causes DNA methylation, plays an important role in the occurrence and development of various tumors. Zhang et al. showed that in breast cancer cell lines treated with paclitaxel, DNMT3a positively regulated LINE-1 mRNA expression levels by increasing methylation within the LINE-1 gene, while promoting the development of a drug-resistant phenotype in breast cancer (83). Therefore, DNA methyltransferase inhibitors (DNMTis) can be used to downregulate DNMT3a levels and thus inhibit LINE-1 expression. Thus, DNA methylation alterations also suggest epigenetics in precision immunotherapy.

#### 5.1.2 Targeted histone modifications

Targeted histone modifications provide another important epigenetic treatment modality. The inhibitory-modified histones (H3K9me3 and H3K9me2) are highly enriched in LINE-1 and silence LINE-1 expression at different stages (84, 85). It has been shown that the histone demethylase KDM4B, a novel regulator of LINE-1, activates the reverse transcriptional activity of LINE-1 by catalyzing the demethylation reaction of the repressive histone H3K9me3 leading to DNA damage, and exhibits increased LINE-1 expression and copy number in breast cancers expressing high levels of KDM4B (86). In addition, hypoxia-induced KDM4B is expressed in approximately 60% of epithelial ovarian cancer (EOC) and positively correlates with the tumor hypoxia marker CA-IX, which is strongly induced in EOC cell lines under hypoxic conditions, and inhibition of KDM4B expression can effectively control ovarian cancer cell invasion and migration *in vitro* (87). Therefore, LINE-1 expression in tumors can be inhibited by using histone demethylase inhibitors. Later, Fukuda et al. identified the H3K9 methyltransferase SETDB1 as a key repressor of retrotransposable elements in mouse embryonic stem cells (mESCs), i.e., histone methylation mediates transcriptional silencing of LINE-1 (88). Taken together, targeting histone demethylation is a major focus of epigenetic therapy for tumors.

#### 5.1.3 Small RNA interference pathway

In addition, the small RNA-mediated pathway governs gene expression in a sequence-dependent manner, inhibiting LINE-1 expression and exerting anti-tumor activity. P-element-induced weak testis protein (PIWI) interacting RNA (piRNA) PIWI-piRNA complex enters the nucleus and targets nascent TE transcripts, recruiting epigenetic silencers, including histone methyltransferases, and even ab initio DNA methylation to maintain transcriptional silencing in the long term (89, 90). RNA interference induced the downregulation of LINE-1 expression in mouse models, leading to a significant reduction in tumorigenicity of cancer cells, and this RNA interference also led to post-transcriptional silencing of LINE-1 elements in human melanoma cells and prostate cancer cells (91). Therefore, RNA interference technology can be considered to reduce the expression level of reverse transcriptase, thus slowing down the proliferation of tumor tissue.

### 5.2 Inhibition of ORF1p activity

The reverse transcriptase (RT) machinery is mainly present within ORF2p, and preclinical studies have shown that RT inhibitors (nevirapine and efavirenz) promote cancer cell differentiation, reduce cell proliferation and antagonize tumor progression in mouse models. Phase II trial species in patients with prostate tumors confirmed the anticancer efficacy of RT inhibitors (91). Furthermore, clinical studies on lung adenocarcinoma have shown that LINE-1 forms a chimeric transcript LCT (L1-FGGY) with the tumor suppressor gene FGGY via reverse transcriptional translocation, and analysis of its function suggests that LCT deletion activates the arachidonic acid (AA) metabolic pathway and promotes tumor growth, which can be effectively targeted by the combination of anti-HIV drugs (NVR) and metabolic inhibitors (ML355) (79). These studies suggest that endogenous RT can be considered as an epigenetic regulator of cell differentiation and proliferation and can be a new target for cancer therapy, and RT inhibitors are effective tools for novel anti-cancer, non-cytotoxic therapy. The chaperone ORF1p also causes loss of retrotransposon activity. Recently, Kou et al. provided new insights into the potential of ORF1p expression inhibitors in antitumor therapy by suggesting that ORF1p may have an independent role in driving tumor development and is considered as a cancer marker (92). They screened LINE-1 ORF1p expression inhibitors in lung cancer cell lines, non-small cell lung adenocarcinoma cell lines, and breast cancer cell lines by efficient high-throughput ICW assays and showed that inhibition of LINE-1 ORF1p expression can affect reverse transcriptional translocation of LINE-1 and inhibit the proliferation of cancer cell lines. These studies point us to the RT and ORF1p encoded by LINE-1 as potential targets in future cancer therapy.

## 6 Discussion

Effective treatment strategies and reliable biomarkers are essential to improve cancer treatment. Currently, among all gynecologic tumors, endometrial and cervical cancers are more often diagnosed at early stages due to different symptoms and screening tools, while ovarian cancer still faces great challenges in its diagnosis and treatment due to the lack of obvious early symptoms. Therefore, the development of effective early diagnostic markers is of great importance for the early detection of cancer. In recent years, The expression of LINE-1 in tumors has aroused particular interest in the scientific community, and numerous studies have shown that LINE-1, as the only autonomously mobile transposon in the human genome, plays an important role in tumorigenesis and development due to its reverse transcriptional activity leading to genomic instability. LINE-1 hypomethylation is an early stage in gynecological tumors such as ovarian, cervical, and endometrial cancers events, and can be used as a molecular marker for early diagnosis and prognosis determination. In addition, ORF1p generated by LINE-1 retrotransposition is highly expressed in ovarian, endometrial, and differentiated vulvar epithelial cancers, and it has been noted that ORF1p is expressed in 90% of HGSOC and 90% of STIC, but is undetectable in 99% of controls; therefore, plasma ORF1p appears to be a highly specific cancer biomarker in gynecologic cancers as well as in high-risk precursor lesions and has important value for tumor prognosis (93). In summary, exploring the function and mechanism of LINE-1 in gynecologic tumors will provide a novel direction for targeted cancer therapy. Its expression in gynecologic tumor tissues such as ovarian cancer, cervical cancer, and endometrial cancer suggests that it is expected to be a novel molecular marker for early diagnosis and prognosis of gynecologic tumors and a potential therapeutic target.

However, the mechanism of LINE-1 in the development of gynecologic tumors has not been fully understood, and few clinical trials have been reported on LINE-1 as a therapeutic target in gynecologic tumors. Therefore, in-depth studies on the mechanism of LINE-1 in gynecologic tumors and related clinical studies are valuable for the early diagnosis of tumors and also help us to find more effective treatments. In conclusion, the use of LINE-1 as a biological agent in gynecologic tumors is of great value. In conclusion, the use of LINE-1 as a biomarker has generated considerable excitement in the scientific community, and it is reasonable to expect that in the coming years, LINE-1 may become a useful biomarker for early diagnosis and prognosis of gynecologic tumors, and research on LINE-1 is expected to become a new strategy for effective treatment of gynecologic tumors.

## Author contributions

JF, TQ, CL, JZ, YD, and MZ performed literature searches. JF, and TQ wrote the first draft of the manuscript. FC, QY, XL, and JZ wrote sections of the manuscript. All authors contributed to the article and approved the submitted version.

## References

[1] Siegel RL Miller KD Fuchs HE Jemal A. Cancer statistics, 2022. CA Cancer J Clin (2022) 72(1):7–33. doi: 10.3322/caac.21708 35020204

[2] Sung H Ferlay J Siegel RL Laversanne M Soerjomataram I Jemal A. Global cancer statistics 2020: GLOBOCAN estimates of incidence and mortality worldwide for 36 cancers in 185 countries. CA: A Cancer J Clin (2021) 71(3):209–49. doi: 10.3322/caac.21660 33538338

[3] Crosbie EJ Kitson SJ McAlpine JN Mukhopadhyay A Powell ME Singh N. Endometrial cancer. Lancet (2022) 399(10333):1412–28. doi: 10.1016/S0140-6736(22)00323-3 35397864

[4] Mills RE Bennett EA Iskow RC Devine SE. Which transposable elements are active in the human genome? Trends Genet (2007) 23(4):183–91. doi: 10.1016/j.tig.2007.02.006 17331616

[5] Hancks DC Kazazian HH Jr. Roles for retrotransposon insertions in human disease. Mob DNA (2016) 7:9. doi: 10.1186/s13100-016-0065-9 27158268 PMC4859970

[6] Burns KH. Transposable elements in cancer. Nat Rev Cancer (2017) 17(7):415–24. doi: 10.1038/nrc.2017.35 28642606

[7] Jonsson ME Ludvik Brattas P Gustafsson C Petri R Yudovich D Pircs K. Activation of neuronal genes via LINE-1 elements upon global DNA demethylation in human neural progenitors. Nat Commun (2019) 10(1):3182. doi: 10.1038/s41467-019-11150-8 31320637 PMC6639357

[8] Goodier JL Kazazian HH Jr. Retrotransposons revisited: the restraint and rehabilitation of parasites. Cell (2008) 135(1):23–35. doi: 10.1016/j.cell.2008.09.022 18854152

[9] Penzkofer T Jager M Figlerowicz M Badge R Mundlos S Robinson PN. L1Base 2: more retrotransposition-active LINE-1s, more mammalian genomes. Nucleic Acids Res (2017) 45(D1):D68–73. doi: 10.1093/nar/gkw925 PMC5210629 27924012

[10] Swergold GD. Identification, characterization, and cell specificity of a human LINE-1 promoter. Mol Cell Biol (1990) 10(12):6718–29. doi: 10.1128/mcb.10.12.6718 PMC362950 1701022

[11] Naufer MN Furano AV Williams MC. Protein-nucleic acid interactions of LINE-1 ORF1p. Semin Cell Dev Biol (2019) 86:140–9. doi: 10.1016/j.semcdb.2018.03.019 PMC6428221 29596909

[12] Mathias SL Scott AF Kazazian HH Jr. Boeke JD Gabriel A. Reverse transcriptase encoded by a human transposable element. Science (1991) 254(5039):1808–10. doi: 10.1126/science.1722352 1722352

[13] Miyoshi T Makino T Moran JV. Poly(ADP-ribose) polymerase 2 recruits replication protein a to sites of LINE-1 integration to facilitate retrotransposition. Mol Cell (2019) 75(6):1286–98 e12. doi: 10.1016/j.molcel.2019.07.018 31473101 PMC6754305

[14] Flasch DA Macia A Sanchez L Ljungman M Heras SR Garcia-Perez JL. Genome-wide *de novo* L1 retrotransposition connects endonuclease activity with replication. Cell (2019) 177(4):837–51 e28. doi: 10.1016/j.cell.2019.02.050 30955886 PMC6558663

[15] Freeman BT Sokolowski M Roy-Engel AM Smither ME Belancio VP. Identification of charged amino acids required for nuclear localization of human L1 ORF1 protein. Mob DNA (2019) 10:20. doi: 10.1186/s13100-019-0159-2 31080522 PMC6501352

[16] Horn AV Celic I Dong C Martirosyan I Han JS. A conserved role for the ESCRT membrane budding complex in LINE retrotransposition. PloS Genet (2017) 13(6):e1006837. doi: 10.1371/journal.pgen.1006837 28586350 PMC5478143

[17] Cost GJ Feng Q Jacquier A Boeke JD. Human L1 element target-primed reverse transcription in vitro. EMBO J (2002) 21(21):5899–910. doi: 10.1093/emboj/cdf592 PMC131089 12411507

[18] Dewannieux M Esnault C Heidmann T. LINE-mediated retrotransposition of marked alu sequences. Nat Genet (2003) 35(1):41–8. doi: 10.1038/ng1223 12897783

[19] Sanchez-Luque FJ Kempen MHC Gerdes P Vargas-Landin DB Richardson SR Troskie RL. LINE-1 evasion of epigenetic repression in humans. Mol Cell (2019) 75(3):590–604 e12. doi: 10.1016/j.molcel.2019.05.024 31230816

[20] Xiong F Wang R Lee JH Li S Chen SF Liao Z. RNA m(6)A modification orchestrates a LINE-1-host interaction that facilitates retrotransposition and contributes to long gene vulnerability. Cell Res (2021) 31(8):861–85. doi: 10.1038/s41422-021-00515-8 PMC8324889 34108665

[21] Hwang SY Jung H Mun S Lee S Park K Baek SC. L1 retrotransposons exploit RNA m(6)A modification as an evolutionary driving force. Nat Commun (2021) 12(1):880. doi: 10.1038/s41467-021-21197-1 33563981 PMC7873242

[22] Sun X Wang X Tang Z Grivainis M Kahler D Yun C. Transcription factor profiling reveals molecular choreography and key regulators of human retrotransposon expression. Proc Natl Acad Sci USA (2018) 115(24):E5526–E35. doi: 10.1073/pnas.1722565115 PMC6004460 29802231

[23] Tiwari B Jones AE Caillet CJ Das S Royer SK Abrams JM. p53 directly represses human LINE1 transposons. Genes Dev (2020) 34(21-22):1439–51. doi: 10.1101/gad.343186.120 PMC7608743 33060137

[24] Mita P Sun X Fenyo D Kahler DJ Li D Agmon N. BRCA1 and s phase DNA repair pathways restrict LINE-1 retrotransposition in human cells. Nat Struct Mol Biol (2020) 27(2):179–91. doi: 10.1038/s41594-020-0374-z PMC7082080 32042152

[25] Liang W Xu J Yuan W Song X Zhang J Wei W. APOBEC3DE inhibits LINE-1 retrotransposition by interacting with ORF1p and influencing LINE reverse transcriptase activity. PloS One (2016) 11(7):e0157220. doi: 10.1371/journal.pone.0157220 27428332 PMC4948907

[26] Briggs EM Mita P Sun X Ha S Vasilyev N Leopold ZR. Unbiased proteomic mapping of the LINE-1 promoter using CRISPR Cas9. Mob DNA (2021) 12(1):21. doi: 10.1186/s13100-021-00249-9 34425899 PMC8381588

[27] Seczynska M Bloor S Cuesta SM Lehner PJ. Genome surveillance by HUSH-mediated silencing of intronless mobile elements. Nature (2022) 601(7893):440–5. doi: 10.1038/s41586-021-04228-1 PMC8770142 34794168

[28] Shademan M Zare K Zahedi M Mosannen Mozaffari H Bagheri Hosseini H Ghaffarzadegan K. Promoter methylation, transcription, and retrotransposition of LINE-1 in colorectal adenomas and adenocarcinomas. Cancer Cell Int (2020) 20:426. doi: 10.1186/s12935-020-01511-5 32905102 PMC7466817

[29] Wang XY Zhang Y Yang N Cheng H Sun YJ. DNMT3a mediates paclitaxel-induced abnormal expression of LINE-1 by increasing the intragenic methylation. Yi Chuan (2020) 42(1):100–11. doi: 10.16288/j.yczz.19-258 31956100

[30] Tunbak H Enriquez-Gasca R Tie CHC Gould PA Mlcochova P Gupta RK. The HUSH complex is a gatekeeper of type I interferon through epigenetic regulation of LINE-1s. Nat Commun (2020) 11(1):5387. doi: 10.1038/s41467-020-19170-5 33144593 PMC7609715

[31] Liu N Lee CH Swigut T Grow E Gu B Bassik MC. Selective silencing of euchromatic L1s revealed by genome-wide screens for L1 regulators. Nature (2018) 553(7687):228–32. doi: 10.1038/nature25179 PMC5774979 29211708

[32] Robbez-Masson L Tie CHC Conde L Tunbak H Husovsky C Tchasovnikarova IA. The HUSH complex cooperates with TRIM28 to repress young retrotransposons and new genes. Genome Res (2018) 28(6):836–45. doi: 10.1101/gr.228171.117 PMC5991525 29728366

[33] Wilson AS Power BE Molloy PL. DNA Hypomethylation and human diseases. Biochim Biophys Acta (2007) 1775(1):138–62. doi: 10.1016/j.bbcan.2006.08.007 17045745

[34] Tapp HS Commane DM Bradburn DM Arasaradnam R Mathers JC Johnson IT. Nutritional factors and gender influence age-related DNA methylation in the human rectal mucosa. Aging Cell (2013) 12(1):148–55. doi: 10.1111/acel.12030 PMC3572581 23157586

[35] Jang HS Shah NM Du AY Dailey ZZ Pehrsson EC Godoy PM. Transposable elements drive widespread expression of oncogenes in human cancers. Nat Genet (2019) 51(4):611–7. doi: 10.1038/s41588-019-0373-3 PMC6443099 30926969

[36] Hoshimoto S Takeuchi H Ono S Sim MS Huynh JL Huang SK. Genome-wide hypomethylation and specific tumor-related gene hypermethylation are associated with esophageal squamous cell carcinoma outcome. J Thorac Oncol (2015) 10(3):509–17. doi: 10.1097/JTO.0000000000000441 25514805

[37] Baba Y Watanabe M Murata A Shigaki H Miyake K Ishimoto T. LINE-1 hypomethylation, DNA copy number alterations, and CDK6 amplification in esophageal squamous cell carcinoma. Clin Cancer Res (2014) 20(5):1114–24. doi: 10.1158/1078-0432.CCR-13-1645 24423610

[38] Kitahara H Okamoto T Shimamatsu S Kohno M Morodomi Y Tagawa T. LINE-1 hypomethylation is associated with malignant traits and cell proliferation in lung adenocarcinoma. Anticancer Res (2020) 40(10):5659–66. doi: 10.21873/anticanres.14579 32988890

[39] Kim Y Rhee YY Wen X Cho NY Bae JM Kim WH. Combination of L1 methylation and tumor-infiltrating lymphocytes as prognostic marker in advanced gastric cancer. Gastric Cancer (2020) 23(3):464–72. doi: 10.1007/s10120-019-01025-8 31691036

[40] Kerachian MA Kerachian M. Long interspersed nucleotide element-1 (LINE-1) methylation in colorectal cancer. Clin Chim Acta (2019) 488:209–14. doi: 10.1016/j.cca.2018.11.018 30445031

[41] Weber B Kimhi S Howard G Eden A Lyko F. Demethylation of a LINE-1 antisense promoter in the cMet locus impairs met signalling through induction of illegitimate transcription. Oncogene (2010) 29(43):5775–84. doi: 10.1038/onc.2010.227 20562909

[42] Harada K Baba Y Ishimoto T Chikamoto A Kosumi K Hayashi H. LINE-1 methylation level and patient prognosis in a database of 208 hepatocellular carcinomas. Ann Surg Oncol (2015) 22(4):1280–7. doi: 10.1245/s10434-014-4134-3 25319577

[43] Ewing AD Smits N Sanchez-Luque FJ Faivre J Brennan PM Richardson SR. Nanopore sequencing enables comprehensive transposable element epigenomic profiling. Mol Cell (2020) 80(5):915–28 e5. doi: 10.1016/j.molcel.2020.10.024 33186547

[44] Park MK Lee JC Lee JW Hwang SJ. Alu cell-free DNA concentration, alu index, and LINE-1 hypomethylation as a cancer predictor. Clin Biochem (2021) 94:67–73. doi: 10.1016/j.clinbiochem.2021.04.021 33901468

[45] Rodic N Sharma R Sharma R Zampella J Dai L Taylor MS. Long interspersed element-1 protein expression is a hallmark of many human cancers. Am J Pathol (2014) 184(5):1280–6. doi: 10.1016/j.ajpath.2014.01.007 PMC4005969 24607009

[46] Ko E-J Oh YL Kim HY Eo WK Kim H Ock MS. Correlation of long interspersed element-1 open reading frame 1 and c-met proto-oncogene protein expression in ovarian cancer. Genes Genomics (2019) 41(11):1293–9. doi: 10.1007/s13258-019-00858-y 31388980

[47] Hosseinnejad K Yin T Gaskins JT Bailen JL Jortani SA. Discovery of the long interspersed nuclear element-1 activation product [Open reading frame-1 (ORF1) protein] in human blood. Clin Chim Acta (2018) 487:228–32. doi: 10.1016/j.cca.2018.09.040 30290158

[48] Whongsiri P Pimratana C Wijitsettakul U Jindatip D Sanpavat A Schulz WA. LINE-1 ORF1 protein is up-regulated by reactive oxygen species and associated with bladder urothelial carcinoma progression. Cancer Genomics Proteomics (2018) 15(2):143–51. doi: 10.21873/cgp.20072 PMC5892605 29496693

[49] Sharp CN Korte EA Hosseinejad K Pitman J Lavasanifar A Eichenberger DJ. ELISA-based detection of open reading frame protein 1 in patients at risk of developing lung cancer. Clin Chim Acta (2020) 507:1–6. doi: 10.1016/j.cca.2020.04.005 32275987

[50] Doucet-O’Hare TT Sharma R Rodic N Anders RA Burns KH Kazazian HH Jr. Somatically acquired LINE-1 insertions in normal esophagus undergo clonal expansion in esophageal squamous cell carcinoma. Hum Mutat (2016) 37(9):942–54. doi: 10.1002/humu.23027 PMC5548391 27319353

[51] Ardeljan D Steranka JP Liu C Li Z Taylor MS Payer LM. Cell fitness screens reveal a conflict between LINE-1 retrotransposition and DNA replication. Nat Struct Mol Biol (2020) 27(2):168–78. doi: 10.1038/s41594-020-0372-1 PMC7080318 32042151

[52] Xia Z Cochrane DR Tessier-Cloutier B Leung S Karnezis AN Cheng AS. Expression of L1 retrotransposon open reading frame protein 1 in gynecologic cancers. Hum Pathol (2019) 92:39–47. doi: 10.1016/j.humpath.2019.06.001 31220479

[53] Harris CR Normart R Yang Q Stevenson E Haffty BG Ganesan S. Association of nuclear localization of a long interspersed nuclear element-1 protein in breast tumors with poor prognostic outcomes. Genes Cancer (2010) 1(2):115–24. doi: 10.1177/1947601909360812 PMC2952938 20948976

[54] Ardeljan D Wang X Oghbaie M Taylor MS Husband D Deshpande V. LINE-1 ORF2p expression is nearly imperceptible in human cancers. Mob DNA (2020) 11:1. doi: 10.1186/s13100-019-0191-2 31892958 PMC6937734

[55] Sciamanna I De Luca C Spadafora C. The reverse transcriptase encoded by LINE-1 retrotransposons in the genesis, progression, and therapy of cancer. Front Chem (2016) 4:6. doi: 10.3389/fchem.2016.00006 26904537 PMC4749692

[56] De Luca C Guadagni F Sinibaldi-Vallebona P Sentinelli S Gallucci M Hoffmann A. Enhanced expression of LINE-1-encoded ORF2 protein in early stages of colon and prostate transformation. Oncotarget (2016) 7(4):4048–61. doi: 10.18632/oncotarget.6767 PMC4826189 26716650

[57] Morse B Rotherg PG South VJ Spandorfer JM Astrin SM. Insertional mutagenesis of the myc locus by a LINE-1 sequence in a human breast carcinoma. Nature (1988) 333(6168):87–90. doi: 10.1038/333087a0 2834650

[58] Wolff EM Byun HM Han HF Sharma S Nichols PW Siegmund KD. Hypomethylation of a LINE-1 promoter activates an alternate transcript of the MET oncogene in bladders with cancer. PloS Genet (2010) 6(4):e1000917. doi: 10.1371/journal.pgen.1000917 20421991 PMC2858672

[59] Zhang R Zhang F Sun Z Liu P Zhang X Ye Y. LINE-1 retrotransposition promotes the development and progression of lung squamous cell carcinoma by disrupting the tumor-suppressor gene FGGY. Cancer Res (2019) 79(17):4453–65. doi: 10.1158/0008-5472.CAN-19-0076 31289132

[60] Miki Y Nishisho I Horii A Miyoshi Y Utsunomiya J Kinzler KW. Disruption of the APC gene by a retrotransposal insertion of LI sequence in a colon cancer. Cancer Res (1992) 52(3):643–5. 1310068

[61] Cajuso T Sulo P Tanskanen T Katainen R Taira A Hanninen UA. Retrotransposon insertions can initiate colorectal cancer and are associated with poor survival. Nat Commun (2019) 10(1):4022. doi: 10.1038/s41467-019-11770-0 31492840 PMC6731219

[62] Rodriguez-Martin B Alvarez EG Baez-Ortega A Zamora J Supek F Demeulemeester J. Pan-cancer analysis of whole genomes identifies driver rearrangements promoted by LINE-1 retrotransposition. Nat Genet (2020) 52(3):306–19. doi: 10.1038/s41588-019-0562-0 PMC7058536 32024998

[63] Nguyen THM Carreira PE Sanchez-Luque FJ Schauer SN Fagg AC Richardson SR. L1 retrotransposon heterogeneity in ovarian tumor cell evolution. Cell Rep (2018) 23(13):3730–40. doi: 10.1016/j.celrep.2018.05.090 29949758

[64] Senthong A Kitkumthorn N Rattanatanyong P Khemapech N Triratanachart S Mutirangura A. Differences in LINE-1 methylation between endometriotic ovarian cyst and endometriosis-associated ovarian cancer. Int J Gynecol Cancer (2014) 24(1):36–42. doi: 10.1097/IGC.0000000000000021 24304685

[65] Curty G Menezes AN Brant AC de Mulder Rougvie M Moreira MAM Soares MA. Expression of retroelements in cervical cancer and their interplay with HPV infection and host gene expression. Cancers (Basel) (2021) 13(14):3513. doi: 10.3390/cancers13143513 34298727 PMC8306386

[66] Lertkhachonsuk R Paiwattananupant K Tantbirojn P Rattanatanyong P Mutirangura A. LINE-1 methylation patterns as a predictor of postmolar gestational trophoblastic neoplasia. BioMed Res Int (2015) 2015:421747. doi: 10.1155/2015/421747 26448937 PMC4584058

[67] McKerrow W Wang X Mendez-Dorantes C Mita P Cao S Grivainis M. LINE-1 expression in cancer correlates with p53 mutation, copy number alteration, and s phase checkpoint. Proc Natl Acad Sci USA (2022) 119(8):e2115999119. doi: 10.1073/pnas.2115999119 35169076 PMC8872788

[68] Hofstetter G Mildner M Tschandl P Pammer J. ORF1p is a potential novel diagnostic marker for differentiated vulvar intraepithelial neoplasia. Int J Gynecol Pathol (2022). 42(2):201–6. doi: 10.1097/PGP.0000000000000907 36044297

[69] Armstrong DK Alvarez RD Bakkum-Gamez JN Barroilhet L Behbakht K Berchuck A. Ovarian cancer, version 2.2020, NCCN clinical practice guidelines in oncology. J Natl Compr Canc Netw (2021) 19(2):191–226. doi: 10.6004/jnccn.2021.0007 33545690

[70] Pattamadilok J Huapai N Rattanatanyong P Vasurattana A Triratanachat S Tresukosol D. LINE-1 hypomethylation level as a potential prognostic factor for epithelial ovarian cancer. Int J Gynecol Cancer (2008) 18(4):711–7. doi: 10.1111/j.1525-1438.2007.01117.x 17944913

[71] Pisanic TR 2nd Asaka S Lin SF Yen TT Sun H Bahadirli-Talbott A. Long interspersed nuclear element 1 retrotransposons become deregulated during the development of ovarian cancer precursor lesions. Am J Pathol (2019) 189(3):513–20. doi: 10.1016/j.ajpath.2018.11.005 PMC6412403 30553834

[72] Tang Z Steranka JP Ma S Grivainis M Rodic N Huang CR. Human transposon insertion profiling: analysis, visualization and identification of somatic LINE-1 insertions in ovarian cancer. Proc Natl Acad Sci USA (2017) 114(5):E733–E40. doi: 10.1073/pnas.1619797114 PMC5293032 28096347

[73] Xia Z Cochrane DR Anglesio MS Wang YK Nazeran T Tessier-Cloutier B. LINE-1 retrotransposon-mediated DNA transductions in endometriosis associated ovarian cancers. Gynecol Oncol (2017) 147(3):642–7. doi: 10.1016/j.ygyno.2017.09.032 29032825

[74] Sato S Gillette M de Santiago PR Kuhn E Burgess M Doucette K. LINE-1 ORF1p as a candidate biomarker in high grade serous ovarian carcinoma. Sci Rep (2023) 13(1):1537. doi: 10.1038/s41598-023-28840-5 36707610 PMC9883229

[75] Yun KY Ko EJ Kim HY Lee JY Eo WK Ock MS. Long interspersed element-1 open reading frame 1 protein expression profiles in ovarian cancers. Genes Genomics (2017) 39(10):1157–62. doi: 10.1007/s13258-017-0589-5 31388980

[76] Smith IM Mydlarz WK Mithani SK Califano JA. DNA Global hypomethylation in squamous cell head and neck cancer associated with smoking, alcohol consumption and stage. Int J Cancer (2007) 121(8):1724–8. doi: 10.1002/ijc.22889 17582607

[77] Barchitta M Quattrocchi A Maugeri A Canto C La Rosa N Cantarella MA. LINE-1 hypermethylation in white blood cell DNA is associated with high-grade cervical intraepithelial neoplasia. BMC Cancer (2017) 17(1):601. doi: 10.1186/s12885-017-3582-0 28854904 PMC5577847

[78] Flatley JE McNeir K Balasubramani L Tidy J Stuart EL Young TA. Folate status and aberrant DNA methylation are associated with HPV infection and cervical pathogenesis. Cancer Epidemiol Biomarkers Prev (2009) 18(10):2782–9. doi: 10.1158/1055-9965.EPI-09-0493 19755648

[79] Sun Z Zhang R Zhang X Sun Y Liu P Francoeur N. LINE-1 promotes tumorigenicity and exacerbates tumor progression via stimulating metabolism reprogramming in non-small cell lung cancer. Mol Cancer (2022) 21(1):147. doi: 10.1186/s12943-022-01618-5 35842613 PMC9288060

[80] Jung H Kim HS Kim JY Sun JM Ahn JS Ahn MJ. DNA Methylation loss promotes immune evasion of tumours with high mutation and copy number load. Nat Commun (2019) 10(1):4278. doi: 10.1038/s41467-019-12159-9 31537801 PMC6753140

[81] Rozek LS Virani S Bellile EL Taylor JMG Sartor MA Zarins KR. Soy isoflavone supplementation increases long interspersed nucleotide element-1 (LINE-1) methylation in head and neck squamous cell carcinoma. Nutr Cancer (2019) 71(5):772–80. doi: 10.1080/01635581.2019.1577981 PMC6513708 30862188

[82] Szabo L Molnar R Tomesz A Deutsch A Darago R Varjas T. Olive oil improves while trans fatty acids further aggravate the hypomethylation of LINE-1 retrotransposon DNA in an environmental carcinogen model. Nutrients (2022) 14(4):908. doi: 10.3390/nu14040908 PMC8878525 35215560

[83] Zhang X Zhang R Yu J. New understanding of the relevant role of LINE-1 retrotransposition in human disease and immune modulation. Front Cell Dev Biol (2020) 8:657. doi: 10.3389/fcell.2020.00657 32850797 PMC7426637

[84] He J Fu X Zhang M He F Li W Abdul MM. Transposable elements are regulated by context-specific patterns of chromatin marks in mouse embryonic stem cells. Nat Commun (2019) 10(1):34. doi: 10.1038/s41467-018-08006-y 30604769 PMC6318327

[85] Walter M Teissandier A Perez-Palacios R Bourc’his D. An epigenetic switch ensures transposon repression upon dynamic loss of DNA methylation in embryonic stem cells. Elife (2016) 5 :e11418. doi: 10.7554/eLife.11418 26814573 PMC4769179

[86] Xiang Y Yan K Zheng Q Ke H Cheng J Xiong W. Histone demethylase KDM4B promotes DNA damage by activating long interspersed nuclear element-1. Cancer Res (2019) 79(1):86–98. doi: 10.1158/0008-5472.CAN-18-1310 30459150

[87] Wilson C Qiu L Hong Y Karnik T Tadros G Mau B. The histone demethylase KDM4B regulates peritoneal seeding of ovarian cancer. Oncogene (2017) 36(18):2565–76. doi: 10.1038/onc.2016.412 PMC5418103 27869162

[88] Fukuda K Shinkai Y. SETDB1-mediated silencing of retroelements. Viruses (2020) 12(6):596. doi: 10.3390/v12060596 32486217 PMC7354471

[89] Ernst C Odom DT Kutter C. The emergence of piRNAs against transposon invasion to preserve mammalian genome integrity. Nat Commun (2017) 8(1):1411. doi: 10.1038/s41467-017-01049-7 29127279 PMC5681665

[90] Zoch A Auchynnikava T Berrens RV Kabayama Y Schopp T Heep M. SPOCD1 is an essential executor of piRNA-directed *de novo* DNA methylation. Nature (2020) 584(7822):635–9. doi: 10.1038/s41586-020-2557-5 PMC7612247 32674113

[91] Sciamanna I Landriscina M Pittoggi C Quirino M Mearelli C Beraldi R. Inhibition of endogenous reverse transcriptase antagonizes human tumor growth. Oncogene (2005) 24(24):3923–31. doi: 10.1038/sj.onc.1208562 15806170

[92] Kou Y Wang S Ma Y Zhang N Zhang Z Liu Q. A high throughput cell-based screen assay for LINE-1 ORF1p expression inhibitors using the in-cell Western technique. Front Pharmacol (2022) 13:881938. doi: 10.3389/fphar.2022.881938 35685648 PMC9171067

[93] Taylor MS Connie W Fridy PC Zhang SJ Senussi Y Wolters JC. Ultrasensitive detection of circulating LINE-1 ORF1p as a specific multi-cancer biomarker. bioRxiv (2023). doi: 10.1101/2023.01.25.525462 PMC10773488 37698949

